# Recent advances in the analysis of metal hyperaccumulation and hypertolerance in plants using proteomics

**DOI:** 10.3389/fpls.2013.00280

**Published:** 2013-07-26

**Authors:** Giovanni DalCorso, Elisa Fasani, Antonella Furini

**Affiliations:** Department of Biotechnology, University of VeronaVerona, Italy

**Keywords:** hyperaccumulator/hypertolerance, heavy metals, proteomics, IEF, abiotic stress

## Abstract

Hyperaccumulator/hypertolerant plant species have evolved strategies allowing them to grow in metal-contaminated soils, where they accumulate high concentrations of heavy metals in their shoots without signs of toxicity. The mechanisms that allow enhanced metal uptake, root-to-shoot translocation and detoxification in these species are not fully understood. Complementary approaches such as transcriptomic-based DNA microarrays and proteomics have recently been used to gain insight into the molecular pathways evolved by metal hyperaccumulator/hypertolerant species. Proteomics has the advantage of focusing on the translated portion of the genome and it allows to analyze complex networks of proteins. This review discusses the recent analysis of metal hyperaccumulator/hypertolerant plant species using proteomics. Changes in photosynthetic proteins, sulfur, and glutathione metabolism, transport, biotic and xenobiotic defenses as well as the differential regulation of proteins involved in signaling and secondary metabolism are discussed in relation to metal hyperaccumulation. We also consider the potential contribution of several proteins to the hyperaccumulation phenotype.

## Introduction

**Figure 1:**
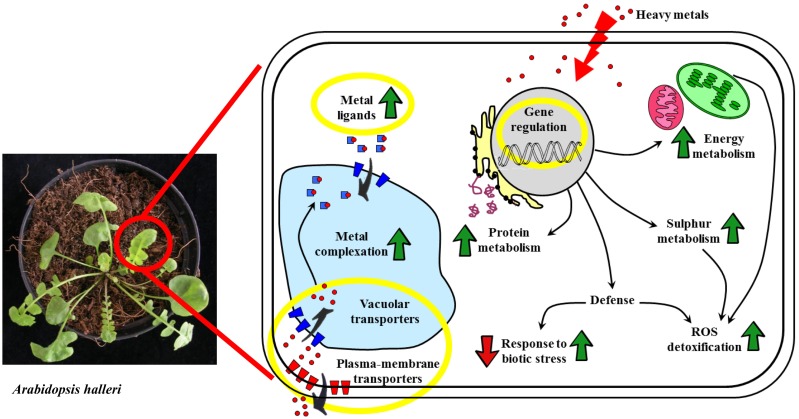
**Schematic representation of the cellular mechanisms responsible for the heavy metal accumulation trait that have been identified through differential proteomics approaches**. Processes that are involved in metal accumulation but whose actors have not been represented in proteomics results are highlighted by yellow circles. Green arrows mean general up-regulation, red arrows mean down-regulation. Red dots: heavy metal ions.

The ability to hyperaccumulate metals in above-ground tissues without phytotoxic effects has evolved in ~500 plant species, mainly those in the Brassicaceae family (Krämer, 2010). Most of these species are Ni hyperaccumulators (Baker et al., 2000). At least three processes make a major contribution to the ability to hyperaccumulate/hypertolerate metals: (1) enhanced root uptake and loading into the xylem; (2) superior root-to-shoot translocation; and (3) efficient detoxification *via* chelation and sequestration, predominantly within leaf cell vacuoles (Clemens et al., 2002). Hyperaccumulators need to regulate their metal homeostasis network precisely. A thorough analysis of the mechanisms involved in metal hyperaccumulation and hypertolerance at the biochemical, genetic and protein levels would provide insight into the corresponding evolutionary and adaptive processes and could be used to develop plants capable of phytoextraction and biofortification. Microarray analysis has been used to compare transcriptional profiles between *Arabidopsis thaliana* (a non-hyperaccumulator species) and two Cd/Zn hyperaccumulator species: *Noccaea caerulescens* (formerly *Thlaspi caerulescens*) and *Arabidopsis halleri* (Becher et al., 2004; Weber et al., 2004; van de Mortel et al., 2006, 2008). These studies indicated that many genes involved in stress responses and metal homeostasis are constitutively expressed at a high level in the hyperaccumulators. The *A. halleri* transcriptome has also been compared to the non-accumulator species *Arabidopsis lyrata* ssp. *petrea*, and to the accumulator and non-accumulator F_3_ hybrid lines derived by crossing them (Filatov et al., 2006). Similarly, transcription profiles have also been compared between *N. caerulescens* ecotypes with different metal tolerance thresholds and uptake capabilities (Plessl et al., 2010). The ability to tolerate and accumulate metals was found to be associated with the ability to cope with reactive oxygen species (ROS), the expression of metal transporters and the suppression of genes involved in defense and disease resistance. In the abovementioned studies, the modulation of gene expression was considered at the level of transcription/mRNA turnover, which may not directly correlate with the protein level, as has been shown for the putative Zn and Mg transporter protein MHX, which is more abundant in *A. halleri* than in *A. thaliana*, even though the corresponding transcript levels are not different (Elbaz et al., 2006). Moreover, RNA analyses do not consider the potential impact of protein folding, stability and localization, protein/protein interactions and post-translational modifications, which are all crucial determinants of protein function. In a proteomic study of the Cd hyperaccumulator *Phytolacca americana*, for instance, the principal sigma factor of the plastidic RNA polymerase, Sig1, which was enhanced upon Cd treatment, seems to have undergone some Cd-induced post-translational modifications (e.g., phosphorylation) that shifted its isoelectric point (Zhao et al., 2011). The proteomic analysis of metal hyperaccumulators is therefore needed to determine the biological functions that arise from changes in gene transcription (Verbruggen et al., 2009). Proteomics not only facilitates the detection of a large number of proteins involved in metal accumulation/detoxification, but also may help to unravel the cross-talk among different pathways. The impact of high metal concentrations on the proteome of hyperaccumulator plants has recently been investigated in detail. In this review, we have focused on recent proteomic approaches that highlight the molecular mechanisms and metabolic activities required for hyperaccumulation and detoxification, particularly the analysis of differentially expressed proteins; a schematic representation is reported in Figure 1. We also discuss the limitations of proteomics in the investigation of hyperaccumulator plants.

## Photosynthesis and energy metabolism

The most recent proteomic experiments show that many of the proteins modulated in hyperaccumulator plants represent the photosynthetic machinery, reflecting the abundance of photosynthetic proteins in green tissues. Interestingly, even though the efficiency of photosynthesis (measured as the photosynthetic quantum yield) is not affected by heavy metals, these proteins tend to be induced by heavy metal treatment (Farinati et al., 2009). In *A. halleri*, Cd and Zn treatment induced the over-accumulation of subunits of antenna systems and photosystems, the cytochrome b_6_/f complex and the ATPase complex (Farinati et al., 2009). Similarly, polypeptide spots corresponding to ribulose bisphosphate carboxylase/oxygenase (RuBisCO), ATPase subunits and oxygen evolving complex (OEC) proteins 1 and 2 accumulated when As was applied to the pseudometallophytes *Agrostis tenuis* (Duquesnoy et al., 2009) and *Pteris vittata* (Bona et al., 2010). In the Zn and Cd hyperaccumulator *Arabis paniculata*, moderate exposure to Zn enhanced the expression of OEC components, whereas excess Zn induced the downregulation of RuBisCO activase thus lowering the efficiency of CO_2_ assimilation (Zeng et al., 2011). The upregulation of photosynthetic proteins under metal exposure might be explained by an increased cellular energy demand, imposed by the hyperaccumulation process. Accordingly, the upregulation of Calvin-Benson cycle enzymes (e.g., RuBisCO, RuBisCO activase, phosphoribulokinase (PRK), and fructose-bisphosphate aldolase) may be necessary to avoid the inhibition of the electron flow through the photosystems (Farinati et al., 2011; Visioli and Marmiroli, 2013). Alternatively, hyperaccumulators usually exhibit strongly enhanced requirements for Zn, even to such an extent that they may suffer from Zn deficiency when growing at Zn supply rates that are considered to be luxurious for non-accumulator plants (van de Mortel et al., 2006). Therefore, the enhanced abundance of photosynthetic proteins, imposed by Zn-exposure, may also be taken to reflect an enhanced scope for growth due to improved Zn nutrition. It is notable that different heavy metals induce different responses. Indeed, although feeble Cd treatments seem to enhance chlorophyll and carotenoid content in the Cd hyperaccumulator *Lonicera japonica* (Jia et al., 2013), in other species, such as the Cd hyperaccumulators *P. americana* and *N. caerulescens*, Cd suppresses photosynthetic proteins and some enzymes of the Calvin-Benson cycle, e.g., RuBisCO and PRK (Hossain and Komatsu, 2012). This has been interpreted as a symptom of Cd toxicity because Zn treatment promotes the activity of RuBisCO and OEC proteins in the same species (Tuomainen et al., 2006; Zhao et al., 2011).

## Sulfur metabolism

Heavy metals have a strong impact on sulfur metabolism because it plays an essential role in metal detoxification, and the modulation of proteins involved in sulfur metabolism has been observed using proteomic approaches in several different hyperaccumulators (Ingle et al., 2005b; Alvarez et al., 2009; Farinati et al., 2009; Bona et al., 2010; Tuomainen et al., 2010; Zeng et al., 2011; Zhao et al., 2011; Schneider et al., 2013). Cysteine biosynthesis is generally induced by heavy metals, e.g., the enzymes serine hydroxymethyltransferase (SHMT, Ingle et al., 2005b), serine acetyltransferase (SAT, Bona et al., 2010) and O-acetylserine(thiol)lyase (OASTL, Ingle et al., 2005b; Bona et al., 2010; Schneider et al., 2013). Interestingly, methionine biosynthesis is suppressed in *Alyssum lesbiacum*, indicating that thiol groups are diverted toward cysteine and glutathione (GSH) biosynthesis (Ingle et al., 2005b). In contrast, methionine synthase was induced after metal treatment in *P. americana* (Zhao et al., 2011), suggesting there are diverse strategies for metal detoxification in hyperaccumulator species. Indeed, methionine is converted into S-adenosylmethionine, which is a precursor in many biosynthetic pathways including nicotianamine (NA) (Higuchi et al., 1999). The cysteine produced in hyperaccumulators enters the GSH biosynthetic pathway, although proteomic analysis has not identified an upregulated enzyme responsible for GSH biosynthesis. However, glutathione-S-transferase (GST) proteins, mainly members of the *phi* class of GST, i.e., GSTFs, were shown to be upregulated upon metal treatment in *N. caerulescens* (Tuomainen et al., 2010; Schneider et al., 2013), *A. lesbiacum* (Ingle et al., 2005b), *A. paniculata* (Zeng et al., 2011), *Brassica juncea* (Alvarez et al., 2009) and *P. americana* (Zhao et al., 2011). GSTFs are involved in the detoxification of xenobiotics by conjugation to GSH (Frova, 2003) and this confirms the role of GSH in metal detoxification not only in the context of ROS scavenging, but also metal ion binding and transport. Furthermore, GSH is the substrate for phytochelatin (PC) biosynthesis, which promotes metal detoxification by chelation. However, PCs are found mainly in non-accumulator species such as *A. thaliana* (Sarry et al., 2006) and *Brassica napus* (Mendoza-Cózatl et al., 2008). Conversely, hyperaccumulator species *A. halleri* and *N. caerulescens* produce very low levels of PCs, although they constitutively express functional phytochelatin synthetase (PCS) genes (Meyer et al., 2011) suggesting they have PC-independent mechanisms for metal sequestration.

## Nitrogen and protein metabolism

The impact of heavy metals on plant metabolism means that metal hypertolerant/hyperaccumulator species must possess mechanisms for more efficient protein turnover. Proteomic analysis has revealed the modulation or specific induction of several proteins involved in protein metabolism in *N. caerulescens* (Tuomainen et al., 2006), *A. lesbiacum* (Ingle et al., 2005b), *A. paniculata* (Zeng et al., 2011), *B. juncea* (Alvarez et al., 2009), *P. americana* (Zhao et al., 2011) and *P. vittata* (Bona et al., 2010). Heavy metals have been shown to affect transcription and translation (Alvarez et al., 2009; Bona et al., 2010; Zeng et al., 2011; Zhao et al., 2011; Visioli et al., 2012), protein folding (Ingle et al., 2005b; Tuomainen et al., 2006; Alvarez et al., 2009; Zeng et al., 2011; Zhao et al., 2011), and protein degradation (Alvarez et al., 2009; Zeng et al., 2011; Visioli et al., 2012). These processes are also induced by metal treatment in non-accumulator species such as *A. thaliana* (Sarry et al., 2006), suggesting a common role in the response to heavy metal stress (Ingle et al., 2005b). However, an essential role in metal accumulation and tolerance is indicated by the differential regulation of proteins involved in protein folding among *N. caerulescens* accessions showing different levels of metal tolerance (Tuomainen et al., 2006) and in Cd-accumulating soybean cultivars (Ahsan et al., 2012). As an alternative explanation, high metal levels may induce increased growth in hyperaccumulator plants characterized by high metal requirement, such as *N. caerulescens* (Shen et al., 1997), leading to enhanced protein metabolism. Higher rates of protein turnover require higher rates of N assimilation, thus explaining the induction of glutamine synthetase in hyperaccumulator species such as *N. caerulescens* (Tuomainen et al., 2006), *A. paniculata* (Zeng et al., 2011) and *P. vittata* (Bona et al., 2011) following exposure to heavy metals to meet the increased demand for nitrogenous compounds.

## Defense-related proteins

Any disruption of the cellular redox status can produce ROS, and plants have evolved a range of mechanisms to detoxify these molecules thus reducing the impact of metal stress. Although hyperaccumulators possess efficient metal chelation systems (Hall, 2002), some also induce the expression of proteins implicated in oxidative defense, e.g., LADH (a putative NADP-dependent oxidoreductase), GST and a mannose-6-phosphate reductase were induced in *A. lesbiacum* after short-term exposure to 0.3 mM NiSO_4_, whereas longer exposure to 0.03 mM NiSO_4_ (sufficient to produce Ni hyperaccumulation in the shoot) did not induce these proteins (Ingle et al., 2005b). Similarly, antioxidant defense proteins such as 2-Cys-peroxiredoxin (2CysPrx) were induced in Cd-treated *P. americana* seedlings. The mature protein 2CysPrx is targeted to the chloroplasts, where it potentially plays a role in the detoxification of H_2_O_2_ (Zhao et al., 2011). Similarly, glutathione peroxidase (GPX), catalase and heat-shock-proteins (HSP) were induced in the fronds of *P. vittata* plants (Bona et al., 2010), whereas iron-superoxide dismutase (Fe-SOD) and glyoxalase I (GLXI) were induced and GSTF2 was down-regulated in *A. halleri* plants treated with Zn and Cd (Farinati et al., 2009). The induction of GST is usually associated with metal stress (Roth et al., 2006), so the suppression of GSTF2 in *A. halleri* suggested that in this hyperaccumulator species symptoms associated with the metal-induced oxidative stress might be alleviated. The inoculation of metal-treated *P. vittata* plants with mycorrhizal fungi and *A. halleri* plants with rhizobacteria suppresses these proteins, suggesting a general improvement of plant fitness due to their interaction with microorganisms (Farinati et al., 2009; Bona et al., 2010). Further analysis showed that ROS scavenging enzymes in *P. vittata* were not detected in the root proteome except for the upregulation of aldehyde dehydrogenase (Bona et al., 2010), a general detoxifying enzyme correlated with stress tolerance (Sunkar et al., 2003). The measurement of protein levels in the leaf epidermal and mesophyll tissues of Zn-hyperaccumulating accessions of *N. caerulescens* revealed that the epidermis adapts to extreme Zn concentrations by accumulating more GST and GSH, ensuring protection against oxidative stress (Schneider et al., 2013). Proteomic analysis revealed that *A. paniculata* expressed different proteins upon Zn or Cd treatment. For instance, Zn treatment induced both energy metabolism and proteins related to stress-scavenging strategies (e.g., ATB2), in the shoot, while xenobiotic/antioxidant strategies were enhanced in Cd-treated plants (Zeng et al., 2011). Interestingly, non-toxic level of heavy metals (beneficial or toxic) can stimulate the growth of several hyperaccumulators and this could be due to both a beneficial effect of improved metal nutrition and/or the activation of mechanisms that are usually deputed to the stress-scavenge. Proteins involved in the jasmonic acid (JA) and salicylic acid (SA) signaling pathways are also modulated in metal hyperaccumulators, e.g., allene oxide cyclase, an essential enzyme in the JA biosynthesis pathway, is downregulated in the shoots of *A. halleri* plants treated with Cd and Zn (Farinati et al., 2009). Under the same conditions, vegetative storage proteins are also strongly suppressed. These proteins function as temporary amino acid stores during plant development, although they also respond to JA (Matthes et al., 2008) and possess acid phosphatase activity that deters insect pests (Liu et al., 2005). High concentrations of metals in *Alyssum bertolonii* (Ernst, 1990) and *N. caerulescens* (Tolrà et al., 2001) are known to reduce the levels of glucosinolates in shoots. Similarly, when *A. halleri* is treated with Cd and Zn, there is a greater than tenfold inhibition in the activity of the β-thioglucoside glucohydrolase, a myrosinase enzyme that breaks down glucosinolates into metabolites that are toxic toward herbivores (Farinati et al., 2009). Furthermore, a β-glucanase with anti-fungal activity was inhibited in *P. vittata* following exposure to As (Bona et al., 2011). Taken together, these results suggest that heavy metal hyperaccumulation downregulates the complex signaling networks that mediate plant defenses against herbivores, insects and pathogens. This supports the controversial hypothesis that metal accumulation in plants provides elemental defense against biotic stress (Boyd et al., 2002; Pollard et al., 2002) which involves cross-talk between heavy metal and defense signaling. On the other hand, proteomic analysis has shown that metals can also induce proteins implicated in biotic stress responses. In *A. thaliana*, several proteomic studies have shown that defense mechanisms activated by pathogens also protect against Cd toxicity (Sels et al., 2008). *N. caerulescens* accessions growing in metalliferous soil express higher levels of β-1,3-glucanase than accessions growing in normal soil (Tuomainen et al., 2010). This pathogenesis-related enzyme is part of the cellulose biosynthesis pathway and may facilitate cell wall restructuring to protect plants from both pathogens and excess metal (van de Mortel et al., 2008). The proteomic analysis of *N. caerulescens* accessions growing in Ni-contaminated soil revealed the induction of an antifungal protein and several defensin-like proteins (Visioli et al., 2012), that are normally induced by pathogens (Carvalho and Gomes, 2009). Defensins are also induced by drought, salinity, cold and signaling through the JA, SA and ethylene pathways (Hanks et al., 2005). The modulation of proteins related to biotic and abiotic stress in hyperaccumulator species and accessions supports their evolutionary plasticity as a result of selection in different environments and genotypic control of the resulting phenotypes.

## Metal ligands

A key metal hyperaccumulation strategy is the ability to chelate metals for detoxification and transport. As discussed above, thiol compounds such as GSH and PCs can bind metals, but only GSH seems to play this role in hyperaccumulator species (Ingle et al., 2005b; Alvarez et al., 2009; Farinati et al., 2009; Tuomainen et al., 2010; Zeng et al., 2011; Zhao et al., 2011; Schneider et al., 2013). NA is the principal ligand for Zn in *A. halleri* (Deinlein et al., 2012) and *N. caerulescens* (Schneider et al., 2013), and transcriptomic studies have confirmed that the nicotianamine synthase (NAS) gene is highly expressed in hyperaccumulators compared to non-accumulators (Becher et al., 2004; Weber et al., 2004; van de Mortel et al., 2006, 2008). Although no modulation of proteins involved in NA biosynthesis was observed in proteomic analysis, methionine biosynthesis is upregulated in *P. americana* following metal treatment (Zhao et al., 2011) and this may be required to increase the NA pool in the presence of heavy metals. The amino acid histidine is the major Ni ligand in *A. lesbiacum* and *N. caerulescens*, and it plays an important role in Ni tolerance and accumulation (Ingle et al., 2005a; Richau et al., 2009). Even so, proteomic analysis has shown that the key enzyme in the histidine biosynthesis pathway (ATP-PRT) is not modulated by metal treatment (Ingle et al., 2005b). Finally, several metallothioneins (MTs), small cysteine-rich metal-binding proteins, are highly expressed in particular ecotypes of *N. caerulescens*. However, high MT expression levels did neither co-segregate with Zn or Cd hyperaccumulation capacity, nor with Zn or Cd tolerance capacity, analyzed in segregating inter-ecotypic crosses, suggesting that these peptides might be primarily involved in Cu homeostasis, rather than Zn or Cd hyperaccumulation/hypertolerance traits as such (Roosens et al., 2004; Hassinen et al., 2009). Proteomic analysis in the same species showed that MT-4C was more abundant in populations characterized by more efficient metal translocation in the shoot (Visioli et al., 2012). It is interesting to note that few proteins responsible for metal ligand biosynthesis have been identified by proteomic analysis, but this does not indicate a lack of involvement in metal tolerance. Indeed, transcriptomic analysis indicates that the corresponding genes may be constitutively expressed at a high level in hyperaccumulators, providing long-term protection against heavy metal stress (Becher et al., 2004; Weber et al., 2004; van de Mortel et al., 2006, 2008).

## Membrane transporters

Membrane proteins are usually classified as integral membrane proteins, characterized by multiple transmembrane regions that anchor the protein into the phospholipid bilayer, or membrane-associated proteins, which are loosely bound to other protein complexes and easily washed away during sample preparation. The standard two-dimensional gel-based 2D proteomic approach for the analysis of complex protein samples involves isoelectric focusing (IEF) followed by sodium dodecylsulfate polyacrylamide gel electrophoresis (SDS-PAGE). Unfortunately, even if characterized by a very high resolution, possibly combinable with quantitative approaches (Rose et al., 2004), IEF is rather poorly compatible with proteomics of integral membrane proteins. In fact, the solubility of membrane proteins is at a minimum when they are at their isoelectric point, resulting in protein precipitation that precludes the following transfer onto the second dimension (Garbis et al., 2005; Speers and Wu, 2007). Strong detergents such as SDS that could solubilize integral membrane proteins are also incompatible with IEF (Speers and Wu, 2007). Because of these inherent difficulties, the importance of membrane-localized metal transporters for metal detoxification and hyperaccumulation was initially determined by transcriptomic analysis followed by protein characterization (Assunção et al., 2001; Becher et al., 2004; Papoyan and Kochian, 2004; Weber et al., 2004). However, gel-based proteomics can be replaced with two-dimensional liquid chromatography/mass spectrometry, which provides a versatile environment suitable for the quantitative analysis of membrane proteins. This approach has identified a P-type ATPase and a ZRT/IRT-like protein (ZIP) transporter in *N. caerulescens* homologous to the *A. thaliana* proteins AtHMA4 and AtZIP4, respectively. In *N. caerulescens*, these were found to be more abundant in the epidermis than in the mesophyll, whereas HMA4 was shown to be confined to the plasma membrane of xylem parenchymatic cells of *A. halleri* (Schneider et al., 2013). This approach also identified NcMTP1 (the *N. caerulescens* homolog of AtZAT/MTP1) which was found to be more abundant, but not enriched, in the epidermis, and NcHMA3, a P-type ATPase that was shown to be significantly enriched in the mesophyll tissue (Schneider et al., 2013). A similar procedure was applied to diverse *N. caerulescens* populations growing in ophiolitic soils, resulting in the identification of ABC27, an ATP binding cassette transporter that was more abundant in the presence of heavy metals and may be involved in the sequestration of metal ions into vacuoles and other subcellular compartments, and in their export across the cell wall (Visioli et al., 2012).

## Concluding remarks: proteomics limitations and challenges

One of the first challenges encountered by plant scientists using proteomics is sample preparation. Reproducibly is essential to ensure that the full complement of proteins in a given sample is captured and separated, and also that operator artifacts and non-proteinaceous contaminants are minimized. Factors such as recalcitrant components (e.g., cellulose and lignin of the cell wall) and plant metabolites (e.g., phenolic compounds, starch and other carbohydrates) often compromise the extraction process, reducing the protein yield and interfering with fractionation and protein separation (Visioli and Marmiroli, 2013). Moreover, the low abundance of key proteins such as membrane transporters and transcription factors, does prevent their detection after sample preparation and protein separation (Garbis et al., 2005). The diverse characteristics of proteins, including size, mass, charge, hydrophobicity, conformational states, post-translational modifications and the formation of complexes, mean that no protocol can accurately recover the entire proteome for a given sample. Such limitations are particularly evident in the case of membrane proteins, as discussed above, excluding the most relevant proteins (trans-membrane transporters) from being represented. To address these challenges, alternative gel-based approaches have been developed in an attempt to maintain the native protein conformation, e.g., Blue Native PAGE uses the dye Coomassie Brilliant Blue G-250 to bind exposed hydrophobic domains thus conferring a net negative charge that enhances electrophoretic mobility and solubilization and thus allows membrane proteins to be separated avoiding precipitation as in IEF (Wittig and Schägger, 2009). Various gel-free approaches have been also developed (Speers and Wu, 2007). Another limitation of differential proteomics is the inability to identify relevant proteins that are constitutively expressed. Even if the abundance of a given protein does not change before and after metal treatment, this does not necessarily indicate that it has no role in metal hyperaccumulation. On the contrary, transcriptomic analysis often shows that genes responsible for hyperaccumulation are strongly and constitutively expressed, suggesting that the hyperaccumulation trait is constitutive and the corresponding proteins are not induced in response to metals but are available all the time (Becher et al., 2004; Weber et al., 2004; van de Mortel et al., 2006, 2008). Therefore, the most informative comparisons are not of hyperaccumulator proteomes before and after exposure to heavy metals, but of proteomes from hyperaccumulator and non-accumulator species/accessions. This strategy has already been tested using the hyperaccumulator *A. lesbiacum* and its non-accumulating close relative *A. montanum*, but the proteomes were too different for meaningful comparison (Ingle et al., 2005b). Comparisons between hyperaccumulator and non-accumulator accessions of the same species may therefore be the most productive way forward to gain insight into the mechanisms of metal hypertolerance and hyperaccumulation. However, even intra-specific comparisons between accessions with contrasting accumulation or tolerance capacities are likely to be confounded by divergent selection under the pressure of factors other than metals themselves. For example, the great majority of proteins that appeared to be differentially expressed among *N. caerulescens* accessions with contrasting accumulation and tolerance capacities, were not differentially expressed among sets of recombinant lines selected for contrasting accumulation and tolerance from inter-accession crosses. Moreover, none of the identified proteins of which the expression level did co-segregate with tolerance or accumulation capacity seemed to have any conceivable role in metal homeostasis (Tuomainen et al., 2010). Therefore, proteomics-based hypotheses concerning the molecular and biochemical mechanisms of hyperaccumulation always need a rigid genetic confirmation.

### Conflict of interest statement

The authors declare that the research was conducted in the absence of any commercial or financial relationships that could be construed as a potential conflict of interest.
